# MALDI imaging mass spectrometry revealed atropine distribution in the ocular tissues and its transit from anterior to posterior regions in the whole-eye of rabbit after topical administration

**DOI:** 10.1371/journal.pone.0211376

**Published:** 2019-01-25

**Authors:** Naoto Mori, Takaharu Mochizuki, Fumiyoshi Yamazaki, Shiro Takei, Hidetoshi Mano, Takeshi Matsugi, Mitsutoshi Setou

**Affiliations:** 1 Nara Research and Development Center, Santen Pharmaceutical Co., Ltd., Ikoma-shi, Nara, Japan; 2 Department of Cellular and Molecular Anatomy, Hamamatsu University School of Medicine, Hamamatsu, Shizuoka, Japan; 3 International Mass Imaging Center, Hamamatsu University School of Medicine, Hamamatsu, Shizuoka, Japan; 4 Laboratory of Fish Biology, Department of Environmental Biology, College of Bioscience and Biotechnology, Chubu University, Kasugai, Aichi, Japan; 5 Department of Systems Molecular Anatomy, Institute for Medical Photonics Research, Preeminent Medical Photonics Education & Research Center, Hamamatsu, Shizuoka, Japan; 6 Department of Anatomy, The university of Hong Kong, Pokfulam, Hong Kong SAR, China; University of Pisa, ITALY

## Abstract

It is essential to elucidate drug distribution in the ocular tissues and drug transit in the eye for ophthalmic pharmaceutical manufacturers. Atropine is a reversible muscarinic receptor used to treat various diseases. However, its distribution in ocular tissues is still incompletely understood. Matrix-assisted laser desorption/ionization–imaging mass spectrometry (MALDI-IMS) evaluates drug distribution in biological samples. However, there have been few investigations of drug distribution in ocular tissues, including whole-eye segments. In the present study, we explored the spatial distribution of atropine in the whole-eye segment by MALDI-IMS, and then evaluated the changes in atropine level along the anterior–posterior and superior–inferior axes. A 1% atropine solution was administered to a rabbit and after 30 min, its eye was enucleated, sectioned, and analyzed by MALDI-IMS. Atropine accumulated primarily in the tear menisci but was found at substantially lower concentrations in the tissue surrounding the conjunctival sacs. Relative differences in atropine levels between the anterior and posterior regions provided insights into the post-instillation behavior of atropine. Atropine signal intensities differed among corneal layers and between the superior and inferior eyeball regions. Differences in signal intensity among tissues indicated that the drug migrated to the posterior regions via a periocular-scleral route. Line scan analysis elucidated atropine transit from the anterior to the posterior region. This information is useful for atropine delivery in the ocular tissues and indicates that MALDI-IMS is effective for revealing drug distribution in whole-eye sections.

## Introduction

Optimization of drug delivery to target ocular tissues remains a challenge for ophthalmic pharmaceutical manufacturers owing to their unique anatomy and physiology. To elucidate the ocular distribution route of particular drugs in animals, we must measure the drug levels in all ocular tissues. Autoradiography (ARG) and drug quantification by high-performance liquid chromatography (HPLC) or liquid chromatography-mass spectrometry (LC-MS) are used to assay drug distribution in ocular tissues. Nevertheless, each of these methods has certain limitations. Preparing radiolabeled compounds with the proper specific activity and radiopurity is costly. Moreover, it may be impossible to distinguish between the parent drug and its metabolites. In HPLC or LC-MS, the eyeball is enucleated and separated into segments including the cornea, conjunctiva, iris/ciliary body, lens, retina, and sclera. Each sample is homogenized with an appropriate solution and the drug concentration in each homogenate is quantified. However, the data collected by this method provide little information about the drug distribution within a specific tissue. There is also a high risk of analyte contamination when the tissue is separated and harvested because certain tissues (aqueous humor, vitreous body, retina, and choroid) are fluid or brittle.

Imaging mass spectrometry (IMS) is a powerful new technique to evaluate drug distribution in biological samples. Also known as mass spectrometric imaging, IMS was first reported by Liebl who used secondary ion mass spectrometry [1]. Since then, its use has rapidly expanded and its application optimized [2,3]. IMS has been used in pharmaceutical research [4,5], drug discovery [6], observation of endogenous molecules (proteins [7], lipids [8], peptides [9], and metabolites [10]), and histopathology [11]. IMS has also been used to analyze the distribution of topically administered benzalkonium chloride in rabbit eye [12]. Benzalkonium chloride penetrated the eye and was detected in both the surface and deep ocular tissues. Hayasaka et al. reported on the identification and spatial distribution of multiple phospholipid species in mouse retina [13]. *N*-retinylidene-*N*-retinylethanolamine (A2E), a component of retinal pigmented epithelial cell lipofuscin, was recently reported in human retina in cases of age-related macular degeneration (AMD) [14]. Therefore, A2E plays a primary role in AMD etiology. However, few studies have reported on drug distribution and transit in ocular tissue, especially in whole-eye sections, using IMS. The eyeball is a complex organ with several barriers against exogenous substances. For this reason, it is difficult to evaluate drug distribution in it. Therapeutic drugs must traverse several organs and barriers to reach the target organ. Therefore, it is necessary to evaluate changes in the quantity of the drug through the transit route. IMS must be able to assess drug transit through the entire eye in order to delineate ophthalmological drug distribution.

In this study, we determined atropine distribution in rabbit ocular tissues by matrix-assisted laser desorption/ionization (MALDI)-IMS. Atropine ophthalmic solution is a widely used reversible muscarinic receptor antagonist. It induces mydriasis by blocking parasympathetic innervation of the pupil and ciliary muscle [15]. It is used to treat iris inflammation and anterior uveitis. It prevents adhesion of the iris to the anterior lens [16]. Atropine may prevent the progression of myopia [17,18]. To our knowledge, little has been reported on atropine distribution in ocular tissues despite its multiple therapeutic applications. Evaluating ocular atropine distribution may help improve understanding of drug efficacy and ocular toxicity. We used IMS to evaluate atropine distribution in a whole-eye section, and then evaluated the changes in atropine level along the anterior–posterior and superior–inferior axes. We also evaluated the effectiveness of MALDI-IMS at revealing drug distribution in whole-eye sections.

## Materials and methods

Our laboratory protocol is deposited in the protocols.io website (dx.doi.org/10.17504/protocols.io.v7ae9ie).

### Chemicals

Atropine sulfate hydrate (special grade), acetonitrile (LC-MS grade), trifluoroacetic acid (HPLC grade), water (LC-MS grade), 2-methylbutane (isopentane), and carboxymethyl cellulose sodium salt (CMC) were purchased from FUJIFILM Wako Pure Chemical Corporation (Osaka, Japan). Acetone was purchased from Nacalai Tesque, Inc. (Kyoto, Japan). Hematoxylin and eosin were purchased from Sakura Finetec Japan Co. Ltd. (Tokyo, Japan). CHCA (α-cyano-4-hydroxycinnamic acid) was purchased from Bruker Daltonik GmbH (Bremen, Germany). The 1% atropine ophthalmic solution was obtained from Nihon Tenganyaku Kenkyusho Co. Ltd. (Nitten, Nagoya, Japan). Somnopentyl (pentobarbital sodium) was obtained from Kyoritsu Seiyaku Corporation (Tokyo, Japan).

### Animal study

Male 24-week-old Japanese white rabbits (Kbl: JW, SPF) were purchased from Kitayama Labs Co. Ltd. (Nagano, Japan). Rabbit is the most common species used for evaluating ocular drug distribution since the rabbit eye is large enough to perform topical drug deliveries. The enzyme atropinesterase, which can rapidly hydrolyze atropine, is present in some rabbits [19]. In order to evaluate atropine distribution without metabolic influence, we investigated atropinesterase activity in rabbit plasma by incubating atropine with plasma in vitro and quantitating atropine concentration. Then we selected rabbits with negative atropinesterase activity. They were housed under a 12-h light-dark cycle (light on at 07h00) room temperature (23 ± 1°C) and a relative humidity of 55 ± 10%. They were given *ad libitum* access to tap water and a gamma ray-sterilized pellet diet (LRC4; Oriental Yeast Co. Ltd., Tokyo, Japan) at ~130 g d^-1^. This study was conducted in accordance with the ARVO Statement for the Use of Animals in Ophthalmic and Vision Research and was approved and monitored by the Institutional Animal Care and Use Committee of Santen Pharmaceutical Co. Ltd. A 1% atropine ophthalmic solution was applied to rabbit eye 3× (50 μL per administration; 5-min intervals). Thirty minutes after the last instillation, the rabbits were euthanized by intravenous injection of 4 mL pentobarbital sodium (64.8 mg mL^-1^). Atropine showed maximum ocular tissue concentration at 30 min after topical administration [20]. Therefore, we set 30 min after the last topical administration as the time point for evaluation.

### Sample preparation for MALDI-IMS analysis

Immediately after the animal was euthanized, its eyeball was excised, washed with saline, and enucleated. Excess saline solution was blotted with a paper towel. The enucleated eyeball was frozen by dipping it in isopentane cooled with dry ice. The frozen eyeball was embedded in pre-cooled 2% CMC solution, frozen in isopentane cooled with dry ice, and stored at −80°C until sectioning. The frozen tissue block was fixed on a stage using optimal cutting temperature compound (Sakura Finetek Japan, Tokyo, Japan) and equilibrated at −20°C on a cryostat (Leica CM1950, Leica Biosystems, Nussloch, Germany). Tissues were sagittally sectioned into 10-μm-thick slices and applied to the surface of an 20-Ω indium/tin oxide-coated glass slide (Matsunami Glass Ind. Ltd., Osaka, Japan). Each slide was dried in a desiccator at room temperature before matrix coating. TMSprayer (HTx Technologies, Chapel Hill, NC, USA), an automated MALDI matrix deposition system, was then used. CHCA (5 mg mL^-1^) in 50% v/v acetonitrile with 0.1% v/v trifluoroacetic acid was sprayed onto the tissue sections using the TMSprayer. After MALDI image acquisition, the matrix was washed off in 100% acetone and the tissue was stained with hematoxylin-eosin.

### MALDI-IMS analysis

Optical images were acquired with an automated image scanner (NanoZoomer Slide Scanner 2.0-HT; Hamamatsu Photonics, Shizuoka, Japan). Mass spectra were acquired with a SolariX FT-ICR mass spectrometer (Bruker Daltonik GmbH, Bremen, Germany) and a 7.0-T superconducting magnet in positive ion mode. Before analysis, the instrument was calibrated with sodium formate. The laser spot size was set to medium focus for low spatial resolution and small focus for high spatial resolution. Its dimensions were ~50 μm and ~25 μm, respectively. Atropine and the CHCA matrix ion were initially detected in the corneal region. The laser intensity was optimized and fixed at the start of each run. The mass range was set to *m*/*z* 200–310. Since far more CHCA sodium adduct (observed *m*/*z*: 212.03) was produced than the CHCA proton adduct (theoretical *m*/*z*: 190.05), the former was set as the lock mass. MALDI-IMS analysis was first performed on the a whole-eye section at a low spatial resolution followed by a high spatial resolution analysis. MALDI mass images were acquired with FlexImaging v. 4.1 (Bruker Daltonik GmbH, Bremen, Germany). Extracted masses were selected within a 0.0009-Da window.

### Data analysis

For the line scan analyses, regions of interests (ROIs) were set at equal distances with FlexImaging v. 4.1 (Bruker Daltonik GmbH, Bremen, Germany). The corneal apex was set to zero (0 mm; S1A Fig). The outer perimeter of each ROI was set to 200 μm and the distance between them was set to 400 μm (S1A and S1B Fig). Each ROI had 2–11 laser spots. Atropine mass spectrum signal intensities were normalized to the total ion current (TIC). The mean atropine mass spectrum signal intensity per ROI was calculated and plotted. The corneal apex was set to zero (center) and used as the graph origin. The Mann-Whitney *U* test was performed between superior and inferior points equidistant from the center. Statistics were run in EXSUS v. 8.0.0 (CAC Croit Corporation, Chuo-ku, Tokyo, Japan). *P* < 0.05 was considered statistically significant.

## Results and discussion

### Atropine mass spectrum by ultrahigh mass resolution MALDI-IMS

**Fig 1:**
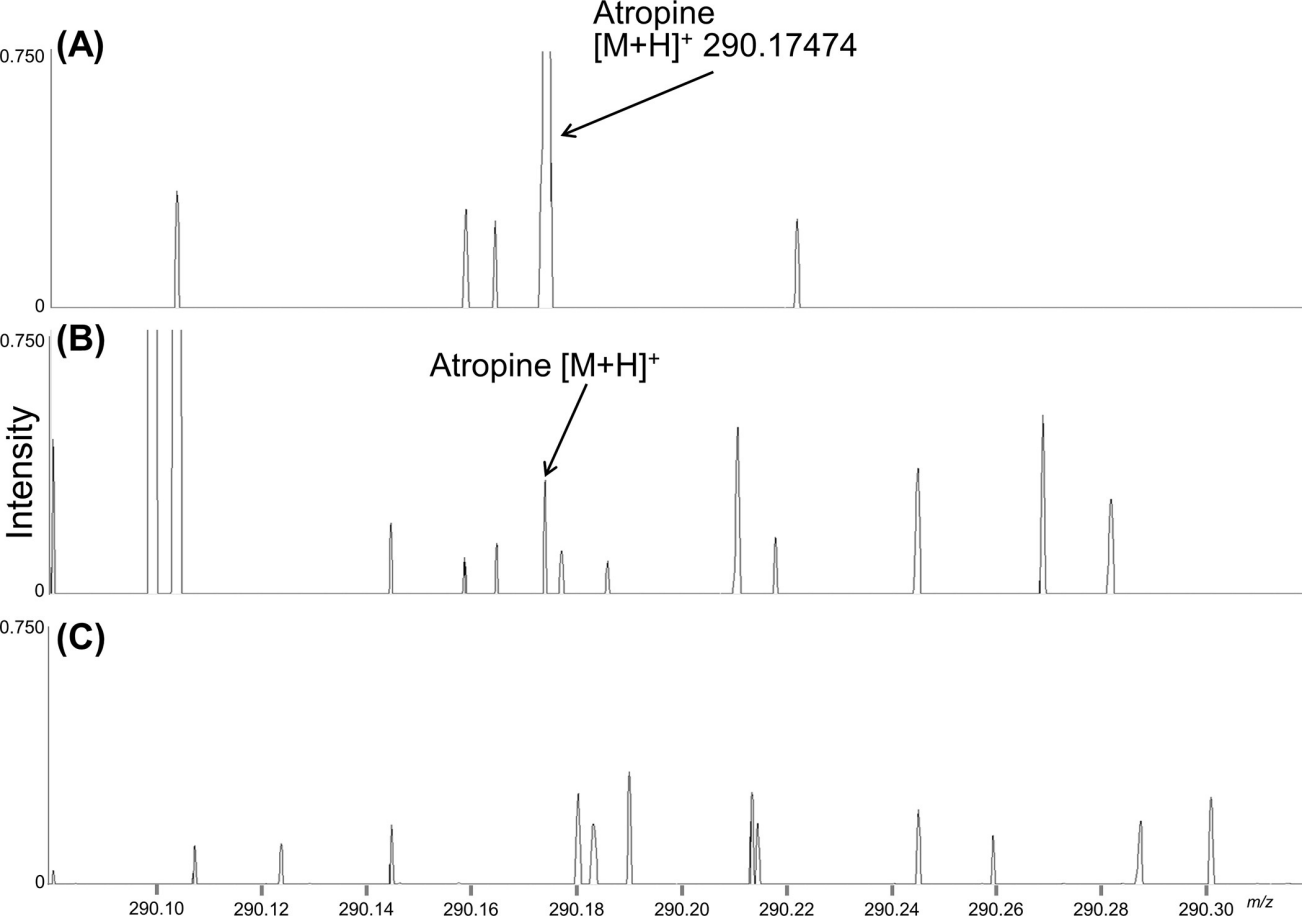
Representative atropine mass spectrum. (A) Mass spectra in the cornea, the back of the eye (B), and the back of the lens (C). The molecular formula of atropine is C_17_H_23_NO_3_ and its mass peak ([M+H]^+^) was observed at *m/z* 290.17474 (theoretical *m/z*: 290.17507).

We first obtained the mass spectrum for corneal atropine since we expected the drug to be abundant there (Fig 1A). The ultra-high mass resolving power allowed the assignment of the atropine peak [atropine + H]^+^ at 290.17474 (theoretical *m*/*z*: 290.17507) with high mass accuracy and clearly separated from all other peaks. Fig 1B shows atropine mass spectrum for the back of the eye. High mass resolution is essential to identify the atropine signal in biological tissues because they contain many other substances in the vicinity of the atropine spectrum, especially in the region where the quantity of the targeted analyte is small. Although the atropine signal was low, it was not superimposed by other endogenous components. Low mass resolution spectrometry may detect atropine and other signals together and misinterpret atropine distribution. Fig 1C shows that the back of the lens had no atropine signal. We normalized the atropine mass spectrum signal intensity to TIC to compare signal intensity in each region. As there is ionization variance due to ion suppression in MALDI-IMS, TIC normalization method is commonly used. It was reported that the distribution data normalized by TIC correlated well with the distribution pattern obtained by MS/MS imaging [21]. Another study reported that TIC normalization improved both qualitative and semi-quantitative ability even among different sections [22]. Some studies reported that internal standard method for quantitative MALDI-IMS [23–25]. However, the primary purpose of this study was not to determine the concentration but to compare the signal intensities among regions and evaluate atropine transit in the whole eye. In addition, we speculated that the internal standard method may have been inappropriate for the whole-eye section. In this method, the internal standard is added to the matrix solution and the tissue/organ section is coated with this mixture. The eyeball is a complex organ. Certain tissues may not be compatible with the matrix solvent due to hydrophilicity or hydrophobicity and result in a heterogeneous matrix (including the internal standard) coating the whole eye which, in turn, causes point-to-point variances in the internal standard signal intensities. Therefore, we chose TIC normalization.

### Atropine distribution in a whole-eye section by MALDI-IMS at low magnification and low spatial resolution

**Fig 2:**
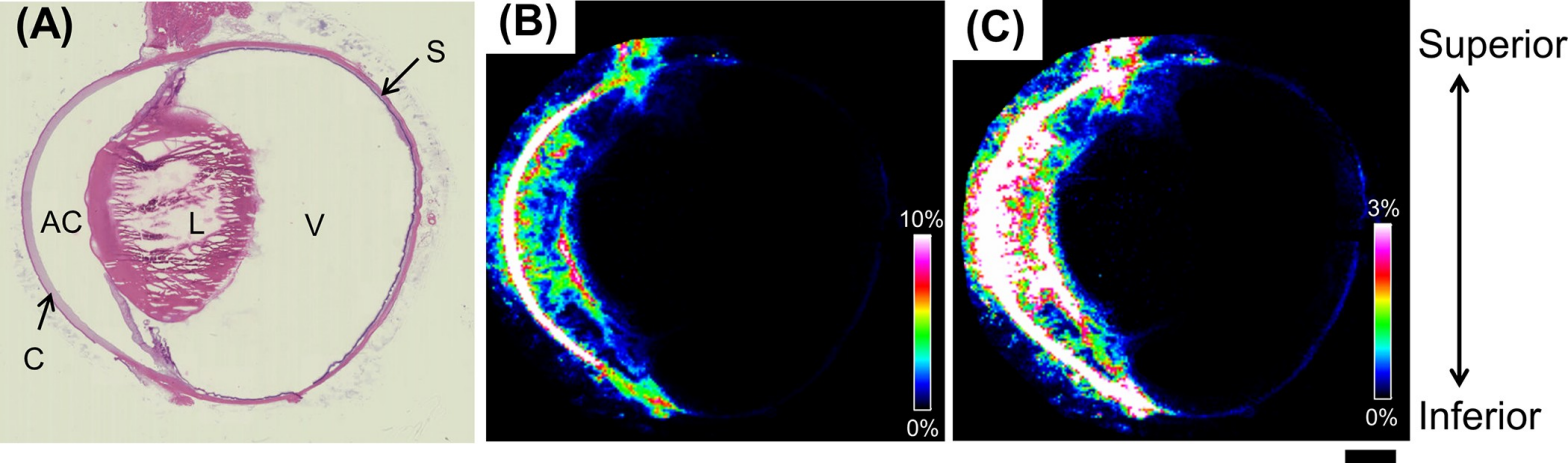
Atropine distribution in a whole-eye section by MALDI-IMS at low magnification and low spatial resolution (100 μm). (A) Optical image of hematoxylin-eosin-stained whole-eye section. (B) Atropine distribution image obtained by IMS. We corrected the image contrast to the anterior segment by adjusting the full-intensity threshold of the result filter. (C) Atropine distribution image obtained by IMS. We corrected the image contrast to the posterior segment by adjusting the full-intensity threshold of the result filter. AC: anterior chamber; L: lens; V: vitreous body; C: cornea; S: sclera. Scale bar = 2 mm.

We examined the atropine distribution at low magnification in a whole-eye section using a 100-μm laser pitch (Fig 2). Fig 2A shows the optical image of a hematoxylin-eosin-stained whole-eye section. Fig 2B and 2C show the atropine distribution acquired by IMS. Large amounts of atropine were distributed in the cornea 30 min after the last instillation. Comparatively less atropine was detected in the anterior chamber, iris, and ciliary body (Fig 2B). We adjusted the full-intensity threshold to reveal the atropine distribution in the posterior region of the eye (Fig 2C). The atropine signal was extremely low in the posterior region, but high mass resolution distinguished between the target mass peak and other endogenous or chemical peaks. High mass resolution lowered the imaging threshold and realized distinct pictures of the posterior region. Atropine was observed in the posterior region but not in the vitreous body or the lens. In the eyeball without atropine administration, no spectra were detected as atropine signals. After topical instillation, the ophthalmic drug reached the posterior segment. Localization of the drug in the posterior region after topical administration is clinically important since a noninvasive method (such as eyedrop application) is needed to treat posterior diseases including AMD, diabetic macular edema, and retinal vein occlusion. All of these are primary causes of vision loss and blindness [26].

### Atropine distribution in a whole-eye section by MALDI-IMS at high magnification and high spatial resolution

**Fig 3:**
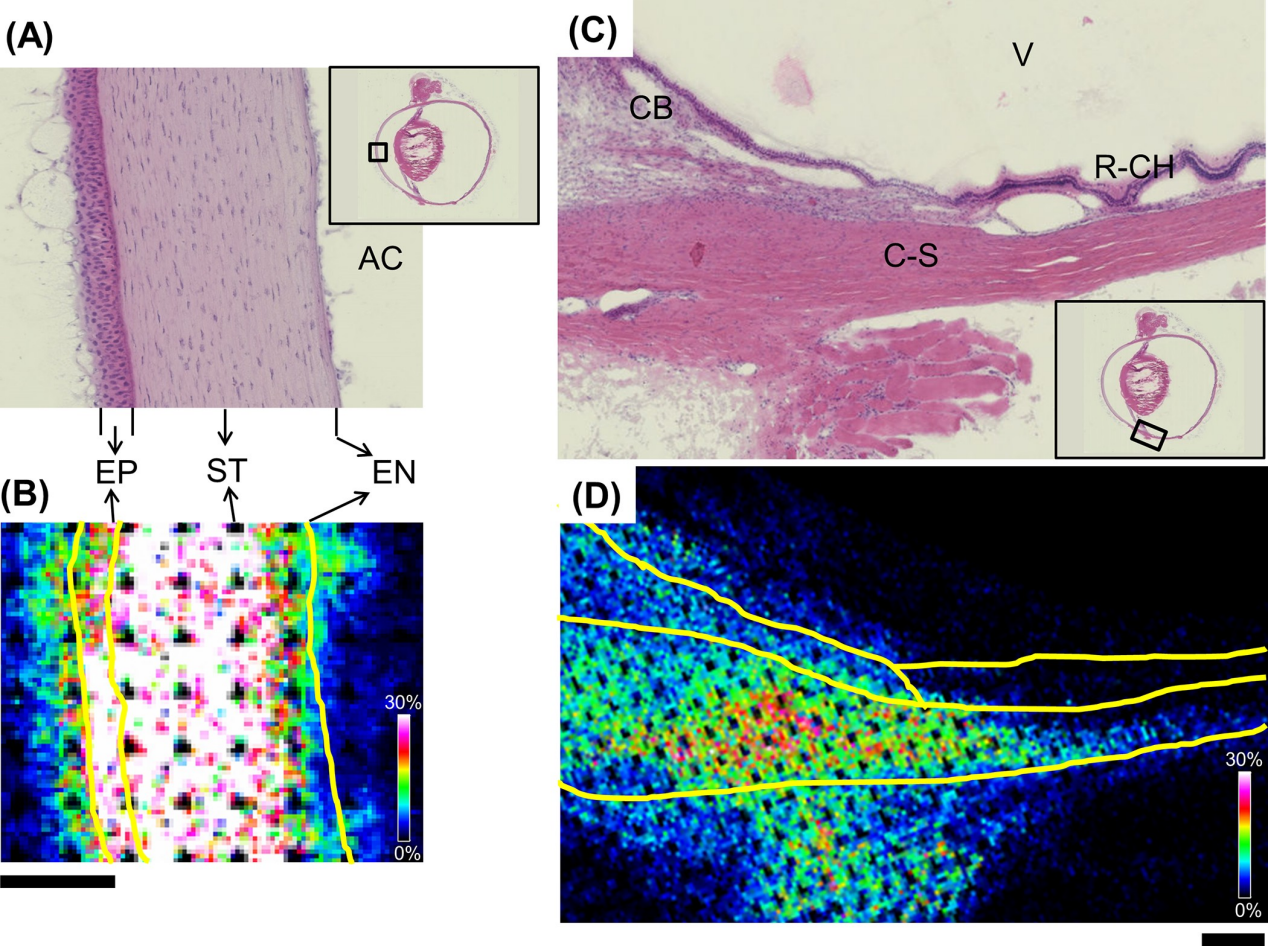
Atropine distribution in a whole-eye section by MALDI-IMS at high magnification and high spatial resolution (10 μm). IMS revealed atropine distribution around the corneal apex (A, B) and in the inferior cornea-sclera junction (C, D). This image was acquired after low spatial resolution (100 μm) imaging analysis (Fig 2). Consequently, data were missing where the sample had been previously irradiated with laser. Yellow lines indicate organ borders. AC: anterior chamber; EP: epithelium; ST: stroma; EN: endothelium; CB: ciliary body; C-S: cornea-sclera; R-CH: retina choroid; V: vitreous body. Scale bar = 200 μm.

We also examined atropine distribution under high spatial resolution. The analysis focused around the center of the cornea (Fig 3A and 3B) and the border of the cornea and sclera (Fig 3C and 3D). These regions are important in terms of drug distribution. The cornea is divided into the epithelium, the stroma, and the endothelium. High spatial resolution provided detailed distribution information about each layer. Signals were high in the epithelium and stroma but low in the endothelium (Fig 3B) possibly because of differences in the structural components and properties of each layer. The corneal epithelium is lipoidal and constitutes 90% of all corneal cells. It creates a significant barrier to the permeation of topically administered hydrophilic drugs [27]. The stroma accounts for ≤90% of the corneal thickness, is composed mainly of hydrated collagen [28], and has no tight junctions. It is a diffusion barrier only to highly lipophilic drugs. The endothelium is also lipoidal but does not offer an important barrier to transcorneal drug diffusion. Prausnitz et al. studied corneal epithelial, stromal, and endothelial permeability [29]. They concluded that the permeability of the endothelium alone to hydrophilic molecules is greater than that of the entire cornea (epithelium, stroma, and endothelium). Therefore, the endothelium is not a rate-limiting barrier. Based on the present and other corneal studies [27–29], we speculated that the hydrophilic atropine readily migrates from the stroma to the aqueous chamber via the endothelium after it penetrates the epithelium.

High-spatial-resolution analysis of atropine distribution along the corneal and scleral margins (Fig 3B and 3D) yielded results similar to those from low-spatial-resolution analysis. Therefore, IMS re-analysis was highly reproducible. The atropine signal was detected in the ciliary body and the cornea-sclera. The signal intensity was lower in the former than the latter. The atropine signal was also weaker in the retina-choroid than the cornea-sclera or ciliary body. Mizuno et al. proposed that topically administered drugs are distributed to the posterior region via the periocular/transposterior-scleral, transvitreal, or uveal route [30]. We found almost no signal in the vitreous body (Figs 2C and 3D). Therefore, atropine was distributed posteriorly through the periocular-scleral and/or uveal but not the transvitreal route. We also detected a low signal intensity in the ciliary body compared to that in the cornea-sclera (Fig 3D). This finding suggests that the main posterior atropine distribution route is periocular-scleral.

### Line scan analysis along the outer circumference of the eyeball section

After topical administration, most of the applied drug is eliminated by tear fluid turnover, blinking, and nasolachrymal drainage. A small amount of atropine is distributed to the ocular tissue.

**Fig 4:**
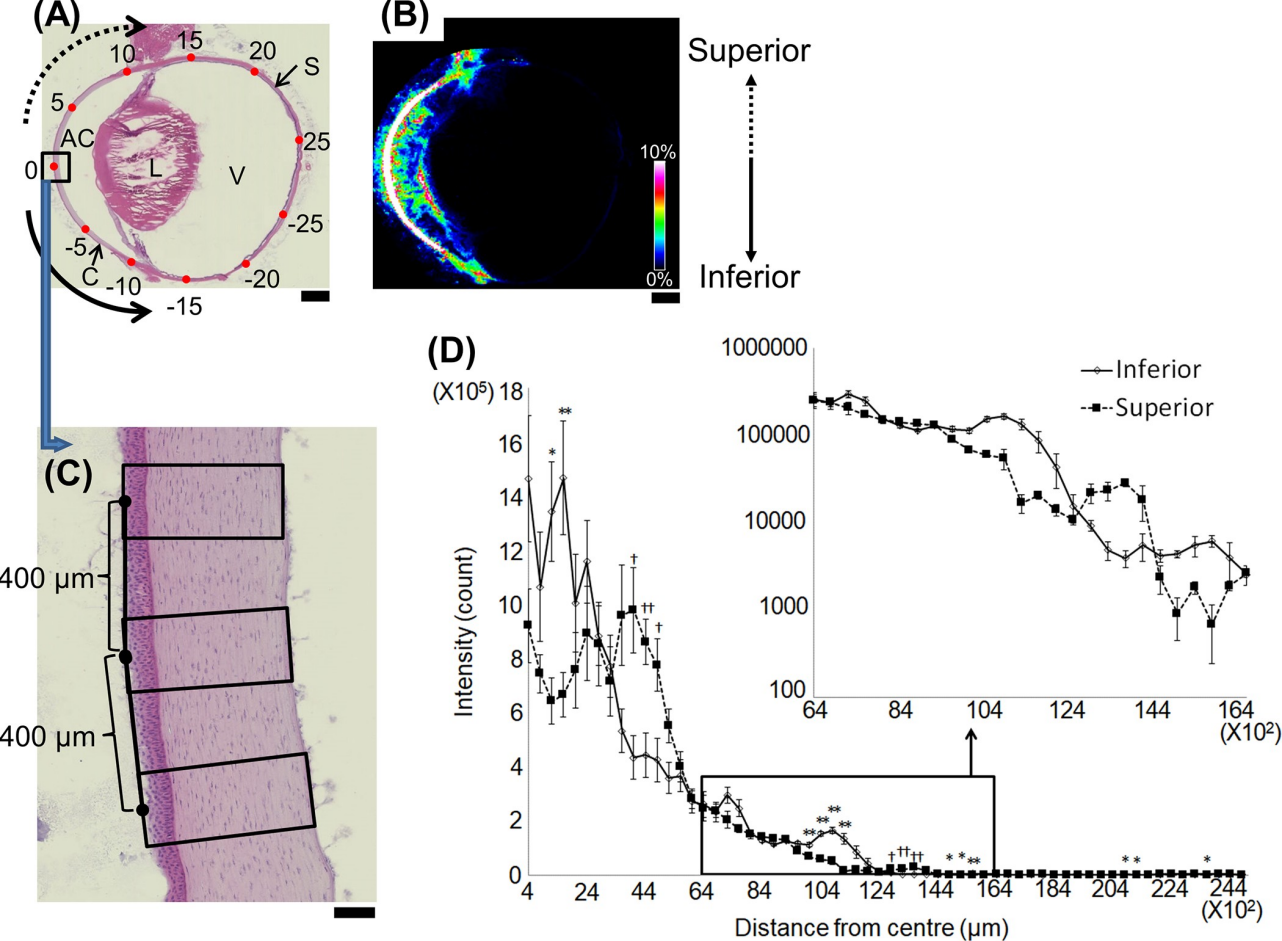
Line scan data along the outer perimeter of the eyeball section. (A) Optical image of a hematoxylin-eosin-stained whole-eye section. Numbers indicate distances (mm) from the zero position. (B) Atropine distribution image obtained by IMS. (C) Magnified segmentation image in the corneal region. The corneal apex (0 mm) was set as the graph origin. (D) Line scan data of the superior (closed square) and inferior (open diamond) halves along the outer perimeter of the eyeball section. Signal intensities were compared between superior and inferior points equidistant from the center. Significantly higher in the superior region: ^†^ *P* < 0.05, ^††^ *P* < 0.01; significantly higher in the inferior region: * *P* < 0.05, ** *P* < 0.01. Mann-Whitney *U* test. AC: anterior chamber; L: lens; V: vitreous body; C: cornea; S: sclera. Scale bars: (A), (B) = 2 mm, (C) = 100 μm.

We analyzed the atropine signal transition from the anterior to the posterior regions (Fig 4). Raw data are available in S1 File. The signal intensity decreased with increasing distance from the corneal apex on both the superior and inferior margins. The signal intensity decreased sharply at ~100 × 10^2^‒140 × 10^2^ μm from the center. The exposed tissue on the outer surface must have come into direct contact with the ophthalmic solution and was, therefore, exposed to large amounts of atropine. However, the tissue in the more posterior area was embedded in the body. Consequently, its exposure to atropine was low.

In the region near the apex where the cornea is exposed to the air (10 × 10^2^‒30 × 10^2^ μm), the signal intensity was higher in the inferior than the superior region. Further away from the apex (30 × 10^2^‒60 × 10^2^ μm), however, the signal intensity was higher in the superior than the inferior region. The positions of the tear menisci may account for these differences. These are located along the edges of the upper and lower eyelids at ~30 × 10^2^‒60×10^2^ μm and 10 × 10^2^‒30 × 10^2^ μm, respectively. The tear menisci are major tear reservoirs. They hold 70‒80% of the tear volume on the exposed ocular surface [31,32]. After topical administration, the ophthalmic solutions immediately mix with the tear film and accumulate primarily in the tear menisci. Shen et al. demonstrated that the lower tear meniscus volume is greater than that of the upper tear meniscus possibly because of gravity [33]. This difference may explain why the signal intensity at 10 × 10^2^‒30 × 10^2^ μm (inferior region) was higher than that at 30 × 10^2^‒60 × 10^2^ μm (superior region).

Signal intensity peaks were observed at 90 × 10^2^‒120 × 10^2^ μm and at 120 × 10^2^‒140 × 10^2^ μm. These locations correspond to the lower and upper conjunctival sacs, respectively. The signal intensity was higher in the inferior region at 90 × 10^2^‒120 × 10^2^ μm and in the superior region at 120 × 10^2^‒140 × 10^2^ μm. This observation may also be the effect of gravity on the eyedrop applied to the eye surface and causing it to accumulate more in the lower than the upper conjunctival sac. Reports on ophthalmic solution fluid dynamics describe this gravitational influence on eyedrops [34]. The concentration of vertically administered ophthalmic drugs is higher in the inferior than the superior regions of the eye. We obtained similar results for eyedrops applied to the rabbit in a vertical position, so our line scan data are reasonable.

### Drug distribution in ocular tissue

To the best of our knowledge, only a few studies on topical atropine distribution in rabbit ocular tissues have been published, including Tigges et al. [35] and Meisner et al. [20]. Tigges et al. applied [^3^H]-atropine solution to rabbit eyes, collected the ocular tissues, and delineated atropine distribution via its radioactivity. One hour after administration, the strongest signal was found in the cornea followed by the sclera, iris/ciliary body, aqueous humor, choroid, retina, and vitreous body. Meisner et al. reported similar results, the strongest signal was found in the cornea followed by the sclera, iris/ciliary body, aqueous humor, lens, and vitreous body at 30 min after topical administration. These results resembled our findings in that the main posterior atropine distribution route was periocular-scleral and indicate that our evaluation method was appropriate.

Another study used IMS to evaluate the distribution of the ophthalmic drug brimonidine in rabbit eyes [36]. A weak drug signal was identified in the posterior portion of the eye. It was proposed that it got there via the periocular-scleral and uveal distribution routes. However, it was difficult to determine the primary transit route in this scenario conclusively because the signal in the posterior region was sparse and the spatial resolution was low (80 μm). Since ocular tissues are complex and small, detailed imaging is necessary to determine drug distribution routes there. Our analysis at high spatial resolution (10 μm) indicated a lower atropine level in the retina-choroid than the cornea-sclera. Therefore, the distribution route was probably periocular-scleral. In addition, the amount change in our line scan analysis confirmed that the atropine reached the posterior region.

IMS with high mass and spatial resolution accurately evaluates atropine distribution in the whole eye. IMS revealed that topically applied atropine localizes at higher levels in the epithelial and stromal layers of the cornea than the endothelium. Therefore, high spatial resolution enables us to perform fine mapping of a drug in the cornea layer. Atropine distribution along the superior and inferior regions of the eyeball was affected by the accumulation of ophthalmic solution. Atropine probably reached the posterior region via the periocular-scleral route. To our knowledge, this is the first study to use IMS and line scan analysis to trace topically administered drug distribution in a whole-eye section with high precision. The high spatial resolution of this method revealed that atropine distributed heterogeneously in the cornea layer. Line scan analysis elucidated atropine transit from the anterior to the posterior region. The target therapeutic effect of the 1% atropine ophthalmic solution used in the experiment was the treatment of mydriasis. It is reasonable to assume that large quantities of atropine are distributed in the iris/ciliary body since atropine blocks parasympathetic innervation of the pupil and ciliary muscles. Atropine may prevent the progression of myopia [17,18]. If we develop an atropine ophthalmic solution to slow the progression of myopia, the formulation must be able to deliver atropine to the sclera [37]. Therefore, we must increase the distribution of the drug to the sclera and decrease its distribution to the iris/ciliary body to reduce side effects. For the purpose of developing a new ophthalmic solution, our analytical method will prove useful by comparing signal intensities between the iris/ciliary body and the sclera after administration. However, since we evaluated distribution at only 30 min after administration, this data is not quantitative. Therefore, further study is needed to elaborate and expand on drug distribution in more detail. To this end, quantitative time-course data is required for ocular atropine distribution.

## Supporting information

S1 Fig ROI setting along the outer perimeter of the eyeball section. (A) Segmentation image of the outer perimeter of the hematoxylin-eosin stained-eyeball section. The corneal apex was set to zero (0 mm). Numbers indicate distances (mm) from the zero position. (B) Magnified segmentation image in the corneal region. The interval of each enclosed region was set to 400 μm. AC: anterior chamber; L: lens; V: vitreous body; C: cornea; S: sclera. Scale bar: (A) 2 mm; (B) 100 μm.
(TIF)

S1 File Line scan data. (XLSX)
