# Supplementary figures and images for: MALDI imaging mass spectrometry revealed atropine distribution in the ocular tissues and its transit from anterior to posterior regions in the whole-eye of rabbit after topical administration
Source: PLoS One. 2019 Jan 25;14(1):e0211376. doi: 10.1371/journal.pone.0211376 (PMC6347209; doi:10.1371/journal.pone.0211376)

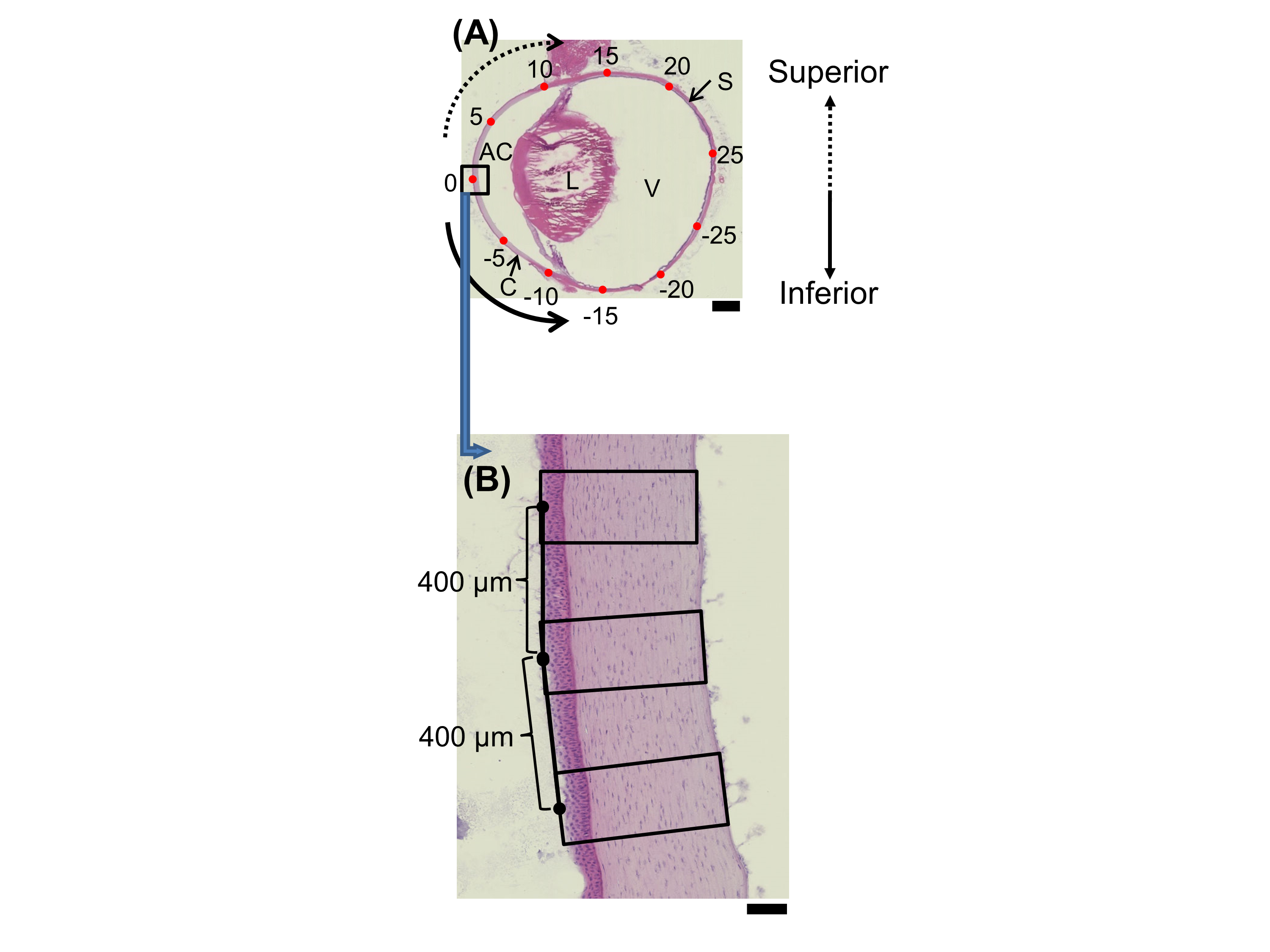

Supplement: S1 Fig — (A) Segmentation image of the outer perimeter of the hematoxylin-eosin stained-eyeball section. The corneal apex was set to zero (0 mm). Numbers indicate distances (mm) from the zero position. (B) Magnified segmentation image in the corneal region. The interval of each enclosed region was set to 400 μm. AC: anterior chamber; L: lens; V: vitreous body; C: cornea; S: sclera. Scale bar: (A) 2 mm; (B) 100 μm. (TIF) [file pone.0211376.s001.tif]
